# Improving sensory integration in Chinese children with moderate sensory integration challenges through engaging basketball training

**DOI:** 10.3389/fpsyg.2024.1481945

**Published:** 2025-01-15

**Authors:** Sha Ge, Xuepeng Guo, Bo Y. Jiang, Alberto Cordova, Jianmin Guan, John Q. Zhang, Wan X. Yao

**Affiliations:** ^1^College of Sports Science, Tianjin Normal University, Tianjin, China; ^2^Department of Physical Education, Tiangong University, Tianjin, China; ^3^School of Public Health, Jilin Medical University, Jilin, China; ^4^College for Health, Community, and Policy, The University of Texas at San Antonio, San Antonio, TX, United States

**Keywords:** sensory integration, sensory integration disorder, sensory integration therapy, basketball, children

## Abstract

**Objective:**

This study aimed to evaluate the effectiveness of combining basketball training with a traditional sensory integration therapy (SIT) vs. a SIT alone in enhancing sensory integration capability among Chinese children diagnosed with mild challenges in sensory integration and sensory processing (CSISP).

**Methods:**

This study comprised a Control group and an Experimental group, both undergoing a 10-week intervention (4 sessions/week, 45 min/session). The Control group exclusively participated in traditional SIT for all sessions. In contrast, the Experimental group engaged in traditional SIT for two sessions per week and Basketball training for the remaining two sessions weekly. Three sensory integration measures (vestibular sensation, tactile perception, proprioception) and five gross motor performance measures (balance beam walking speed, single-leg standing with eyes closed, tennis ball throw, two-legged jump speed, 10-m shuttle run) were assessed before and after the 10-week training period. Separate 2 (Group) × 2 (Test Phases) Analysis of Variance (ANOVA) with repeated measures on the second factor were conducted for each measure. Interaction effects were further explored using Tukey's HSD test to clarify their nature.

**Results:**

Both groups demonstrated significant improvements in all sensory integration and gross motor performance measures following the 10-week training sessions (*p* < 0.001). Importantly, the group receiving the combination of basketball training and traditional SIT significantly outperformed the group undergoing SIT alone in all assessed areas.

**Conclusion:**

These results indicate that combining basketball training with traditional SIT enhances sensory integration capabilities more effectively among Chinese children aged 4–6 years with moderate CSISP compared to utilizing SIT alone.

## Introduction

Sensory Integration refers to the process by which the brain organizes and interprets sensory information from the environment and the body to produce appropriate responses. This complex function involves integrating sensory inputs from various modalities—such as vision, hearing, touch (encompassing awareness of touch, pressure, vibration, and temperature on the skin's surface), taste, smell, vestibular (involving awareness of balance, spatial orientation, and head movement), and proprioception (involving awareness of body position, movement, and muscle tension)—to help individuals make sense of their surroundings and interact effectively with their environment. The foundational concepts of sensory integration began to emerge in the early 20th century. However, it was not until the work of A. Jean Ayres, an American occupational therapist and psychologist, that the theory was formally defined. Ayres started developing the theory of sensory integration in the 1960s and 1970s, with key publications such as “Sensory integrative processes and neuropsychological learning disability” (Ayres, 1968) and “Sensory integration and learning disorders” (Ayres, 1972a). Ayres proposed that difficulties in processing and organizing sensory information could lead to various behavioral and developmental challenges.

The development of an individual's motor, sensory, and cognitive abilities occurs simultaneously with brain maturation (Johnson, 2005). As the cerebral cortex matures, it gradually integrates external information, giving rise to a complex process known as “sensorimotor-cognitive function.” Over time, individuals gain a comprehensive understanding of their environment and acquire the ability to regulate their behavior effectively. Sensory integration, as described by Ayres (1972a), occurs when the brain fully analyzes and processes information from both the environment and different parts of the body, making decisions that enable smooth activity. However, during development, the sensory integration may become dysfunctional, resulting in challenges in sensory integration and sensory processing (CSISP) due to inefficient signaling or functioning of sensory neurons. These disorders can lead to deficits in both cognitive and motor performances (Ayres, 1972b; Zimmer and Desch, 2012).

Children with CSISP can exhibit a range of symptoms across various sensory modalities, significantly affecting their daily functioning, behavior, and interactions. Major symptoms typically include sensory over-responsivity, sensory under-responsivity, sensory modulation difficulties, sensory discrimination problem, motor coordination issues, behavioral and emotional symptoms, and impacts on social and cognitive development (Ayres, 2005; Mielnick, 2017). Research findings indicate that the prevalence of children with CSISP in the United States ranges from 5 to 20% (Ahn et al., 2004; Kong and Moreno, 2018; Nielsen et al., 2021). In contrast, the rate is notably higher in China, with estimates ranging from 29% to 35% of children affected by CSISP (Ren et al., 1995; Xie et al., 2012). Studies have shown that various factors can influence the development of CSISP, including unstable maternal emotional status caused by a noisy living environment and exposure to alcohol and cigarettes during pregnancy (Yuan et al., 2009), as well as limited access to physical activity facilities (Li et al., 2007), and lower social-ecological status within families (Li et al., 2007; Wang et al., 2012). Hence, the significant disparity in CSISP prevalence between the two countries could potentially stem from differences in living environments and exposure to such factors during pregnancy.

In addition to the aforementioned symptoms, it is imperative to emphasize that children with sensory integration challenges often exhibit reduced participation in educational activities and diminished academic performance (Bar-Shalita et al., 2008; Koenig and Rudney, 2010). Therefore, finding effective interventions to tackle these disorders is of utmost importance. Sensory-Integration Therapy (SIT), initially developed by Ayres (1972b, 1979), is a prominent intervention used in both the United States and China to address children affected by sensory integration disorders. In China, there has been a growing focus on exploring the efficacy of integrating sport activities into SIT.

For instance, Xu et al. (2019) combined Taekwondo with Sensory Integration Therapy and found that after 12 weeks of Taekwondo training, there was a beneficial effect on vestibular, proprioceptive, tactile functions, and learning abilities in children aged 6–10 with sensory integration disorders. This combination approach facilitated the development of sensory integration in these children and improved their sensory integration abilities. Similarly, Han (2014) implemented a 12-week martial arts intervention for the children with CSISP. The results indicated that martial arts had a significant impact on proprioceptive and vestibular functions, although it had a minimal effect on tactile defensiveness. Additionally, martial arts positively influenced children's balance and coordination. Zhao (2020) conducted a 12-week program of rhythmic rope skipping with second-grade elementary school children. The study found that rhythmic rope skipping had a very significant effect on children's proprioceptive and tactile functions, and it also led to significant changes in their vestibular functions. Additionally, Ge et al. (2019) incorporated cheerleading into the daily activities of preschool children, conducting a 10-week cheerleading training program for children aged 4–6. After the 10 weeks, there were significant improvements in various abilities of the children. The study indicated that 10 weeks of cheerleading training could address issues related to sensory integration dysfunction in young children. Overall, these studies consistently indicates that the combination of traditional SIT with sport activities yields superior outcomes compared to employing SIT alone in treating CSISP.

It is noteworthy that the aforementioned sports are primarily individual events and are classified as closed motor skills. Research has shown that open motor skills are more effective than closed motor skills in enhancing children's executive function (Feng et al., 2023; Heilmann et al., 2022). Motor skills can be categorized based on the stability of the environmental context in which they are performed, resulting in two main types: closed motor skills and open motor skills (Magill and Anderson, 2021).

Closed motor skills are performed in a stable environment where the supporting surface, objects, and other people involved remain stationary throughout the execution of the skill. This stability allows the performer to initiate movements at their own pace, which is why closed motor skills are often referred to as self-paced skills. Gymnastic and track-and-field events are typical examples of closed motor skills, as the environmental context does not change during performance. In contrast, open motor skills occur in dynamic environments where the supporting surface, objects, and/or other people are in motion. To successfully execute open motor skills, performers must adapt their actions in response to these moving elements. This adaptation requires timing the initiation of movements according to the changes in the external environment, which is why open motor skills are sometimes termed externally-paced skills (Magill and Anderson, 2021).

As highlighted in systematic reviews (Feng et al., 2023; Heilmann et al., 2022) suggests that that open motor skills (e.g., basketball, wrestling, fencing) provide greater benefits than close motor skills (e.g., running, swimming) for certain executive functions, such as cognitive flexibility and working memory. In a study comparing aerobic training (i.e., treadmill run, a closed motor skill) and designed-sport training (i.e., free-style wrestling, an open motor skill), Moreau et al. (2015) found that after eight-weeks training, the wrestling group demonstrated the most significant improvements in working memory-related measures and spatial awareness, underscoring the effectiveness of open motor skills in enhancing cognitive performance. Additionally, the wrestling training was associated with notable health benefits, including reductions in heart rate and blood pressure. Similarly, Gökçe et al. (2021) compared the long-term effects of participation in open-skill (i.e., fencing) vs. closed-skill (i.e., swimming) exercises on cognitive functions and levels of Brain-Derived Neurotrophic Factor (BDNF). Their findings indicated that fencers outperformed swimmers and sedentary controls on tasks measuring visuospatial working memory, verbal fluency, and selective attention. This further supports the unique cognitive advantages associated with open motor skills.

In summary, these studies consistently demonstrate that open-motor-skill training offers superior cognitive gains compared to closed-motor-skill training, emphasizing its potential for enhancing both cognitive and physical health outcomes.

Basketball falls into the category of open motor skills. The sport involves a variety of dynamic actions, such as sprinting, jumping, quick directional changes, dribbling, throwing, and catching. These activities require players to constantly adjust their movements based on the actions of teammates, opponents, and the ball, thereby enhancing their ability to respond to a changing environment.

Furthermore, basketball as a team sport offers substantial opportunities for children to develop communication skills through verbal and non-verbal cues with peers. This interaction is particularly beneficial for children with CSISP, as it helps build social skills and teamwork. A systematic review (Eime et al., 2013) revealed that children and adolescents who played team sports felt greater self-esteem, less fearful in social situations, less socially isolated and more socially accepted compared to their peers who participated in individual sports or no sport. It is worth to note that longitudinal studies indicates that team sport participation was found to be less depressed (Babiss and Gangwisch, 2009), greater self-esteem for females (Pedersen and Seidman, 2004), and to be associated with lower social isolation later in life (Barber et al., 2001).

The dynamic nature of basketball may potentially make it effective for enhancing vestibular, tactile, and proprioceptive functions. The sport's requirements for speed, power, and eye-hand coordination significantly contribute to the development of these sensory and motor skills. Considering these features and basketball's widespread popularity in both China and around the world, it was chosen as a key component of the study to evaluate its combined efficacy with traditional sensory integration therapy.

The primary aim of this study was to assess whether integrating basketball training with traditional SIT would produce superior results in managing CSISP compared to SIT alone. It was hypothesized that the combined approach would lead to more significant improvements in reducing CSISP symptoms and enhancing overall therapeutic outcomes.

## Research methods

### Participants

A total of 40 children, aged between 4 to 6 years with moderate CSISP, participated in the study. Specifically, 20 children were randomly assigned to the experimental group, while an additional 20 children were allocated to the control group. All participants were recruited from local preschools. The study was approved by the Ethics Committee of Tianjin Normal University, China (Approval number: 2023122902) on December 29th, 2023. Furthermore, parents or guardians of all participants provided written consent before the children participated in the study. It's important to note that all participants were free from any other current or past neurological or physical impairments known to affect neuronal and motor conduction, except for moderate CSISP.

All participants were selected based on the following inclusion criteria:

1) Moderate CSISP. CSISP was determined by using a Chinese version of the Child Sensory Integration Scale (see more detailed description of the instrument below).
2) No prior experience in the intervention conducted in the current study.
3) Right-handedness, as determined by the Edinburg Handedness Inventory test (Oldfield, 1971). Previous research (Darvik et al., 2018) has shown that left-handedness prevalence may adversely affect developmental motor coordination. Therefore, to mitigate any potential influence of handedness on intervention outcomes, the current study exclusively enrolled right-handed participants.
4) No participation in any other intensive sport/motor activities immediately before and during the experimental period.
5) A computer-generated randomization schedule was used to assign eligible participants to either the experimental or control group. Randomization ensured unbiased allocation, and groups were balanced to minimize variability in key demographic and clinical characteristics, such as age and CSISP severity.

See Table 1 for the demographic characteristics of the participants.

**Table 1:** Participants' demographic characteristics.

	**Age**	**Weight**	**Height**
**Control group**			
Male (*n* = 10)	5.20 ± 1.03	23.42 ± 3.52	127.08 ± 5.84
Female (*n* = 10)	5.10 ± 1.10	22.89 ± 3.80	122.65 ± 5.94
**Experimental group**			
Male (*n* = 10)	5.20 ± 1.03	23.23 ± 3.09	122.71 ± 4.72
Female (*n* = 10)	5.10 ± 1.10	23.03 ± 2.41	126.80 ± 5.86

### Sensory integration measurement

Sensory integration was evaluated using the Child Sensory Integration Scale, a questionnaire originally developed by Ayres (2005) and revised by Ren et al. (1994) to fit Chinese cultural population. This scale has demonstrated acceptable reliability, with re-test reliability ranging from 0.47 to 0.73 and split-half reliability from 0.68 to 0.77. Factor analysis confirmed its structural validity, showing strong correlations (0.49–0.94) between extracted factors and individual items. Cronbach's alpha values ranged from 0.44 to 0.63 (*p* < 0.01), indicating sufficient construct validity. Consequently, this revised version of Child Sensory Integration Scale has been widely adopted in mainland of China (Deng et al., 2023; Fu et al., 2022).

The scale is divided into five aspects: vestibular sensation, tactile perception, proprioception, learning ability, and special issues related to children aged 10 years or older. Since the current study focused on children aged between 4 and 6 years and motor-related behaviors, only the first three aspects of the scale were included in the study:

1. Vestibular sensation: this aspect encompasses 14 items focusing on issues such as clumsiness with hands and feet, and being prone to falls.
2. Tactile perception: this component encompasses 21 items that assess emotional stability and a propensity for over-defense. It focuses on issues such as shyness, discomfort, a preference for solitude, reluctance to socialize, sensitivity to stimuli from TV or stories, and tendencies to react with sudden outbursts of shouting or laughing.
3. Proprioception: this component includes 12 items that assess the body's sense of proprioception and balance coordination. It addresses issues such as slow performance in tasks like dressing and tying shoelaces, aversion to activities such as somersaults, rolling over, and climbing heights.

The scale uses a five-level rating system: “Never, Rarely, Sometimes, Often, Always,” with scores of 5, 4, 3, 2, and 1, respectively.

The raw scores are converted into standardized *T*-scores by using following formula (Ren et al., 1994):


$$\begin{array}{l} T_{i}=20+15^{*}\left(X_{i}-\bar{X}\right)/SD, \end{array}$$

*where T*_*i*_ *is the T-score for the ith individual, “20” is the intercept, “15” is the slop, X*_*i*_ *is the ith individual's raw score, X is the group mean, and SD is the standard deviation*.

Standard *T*-scores ≥ 40 indicate normalcy. Scores between 30 and 40 indicate mild sensory integration dysfunction, scores between 20 and 30 indicate moderate sensory integration dysfunction, and scores ≤ 20 indicate severe sensory integration dysfunction.

The questionnaire was completed by parents and/or guardians. A total of 276 questionnaires were collected, with a response rate of 94.52%. After the exclusion of the incomplete ones, 204 valid questionnaires remained, including 100 with mild or no sensory integration dysfunction, 82 with moderate dysfunction, and 22 with severe dysfunction. The 40 participants (20 for the experimental group, 20 for the control group) were randomly selected from the 82 preschoolers with moderate sensory integration dysfunction.

### Gross motor performance measurement

Gross motor performance was assessed using the Chinese National Physical Fitness Measurement Standards (Revised in 2023), which encompasses the domains of balance, coordination, and agility.

1. Balance ability:

1) Balance Beam Walking Speed: the time taken to pass through a 5-m long balance beam was recorded. Participants must concentrate while standing on one foot on the beam, with both arms held out to the sides. They then stepped forward with the other foot onto the beam and alternate feet while moving forward.
2) Single-leg standing with eyes closed: the time from lifting one foot off the ground to both feet touching the ground again was recorded while the participant stands with their eyes closed.

2. Coordination ability:

1) Tennis ball throw: participants threw a tennis ball while standing in place, and the distance of the throw was measured and recorded.
2) Two-legged jump speed: the children will put their feet together, fold their hands behind their backs, bend their legs forward at a 45-degree angle, and jump forward, covering a distance of 8 m while recording the time.

3. Agility: 10-m shuttle run: participants ran back and forth within a 10-m zone, and the time taken to complete the run was recorded.

Each participant had two opportunities for each test, and the better of the two was used for further data analysis.

### Blinding procedures

To minimize potential bias, all data collectors and raters were blinded to group assignments. Participants were anonymized with identification codes, and assessors were not informed of the intervention conditions assigned to each group. This ensured impartiality in evaluating sensory integration and gross motor skills, particularly for subjective assessments.

Additionally, to ensure intervention fidelity, a detailed monitoring system was implemented. Interventionists completed session checklists, and trained supervisors conducted regular observations to ensure adherence to the protocol. Any deviations were promptly addressed, maintaining the integrity of the intervention.

### Intervention protocols

#### Intervention programs

Two intervention programs were implemented in the study: traditional sensory integration training (SIT) and Basketball training.

1) The SIT program: The SIT program followed the guidelines described in *Illustrated Children's Sensory Integration Training* (Li, 2018), which was developed based on the original SIT program established by Ayres (1972b, 1979). This training encompasses 20 items focusing on developing balance and coordination abilities (see Supplementary material A for details of the program).
2) Basketball program: the Basketball program was designed by the experimenters in consultation with experts in the field of CSISP and local kindergarten teachers. This program consists of 20 items and focuses on developing fundamental basketball skills through participation in game-oriented basketball fundamental skill practices (see Supplementary material B for details of the program).

#### Intervention design

The interventions were conducted at a local training camp for children aged from 4 to 6 years. This study comprised two groups: a Control group and an Experimental group. Both groups participated in a 10-week intervention, with four sessions per week, each lasting 45 min. The Control group engaged solely in traditional SIT for all sessions. In contrast, the Experimental group participated in traditional SIT for two sessions per week and Basketball training for the other two sessions each week.

During the 10-week intervention period, parents and/or guardians were instructed to ensure that the children did not engage in any other intensive motor-related activities.

### Statistical analysis

The dependent variables in the two categories (Sensory Integration and Gross Motor Performance) were analyzed using separate 2 (Control group vs. Experimental group) × 2 (Test phase: pre-intervention vs. post-intervention) ANVOAs with repeated measures on the second factor. The significant level was set at *p* £ 0.05. If any interaction effects were found, further analyses were conducted using Tukey's HSD test to explore the nature of these interactions.

## Results

Following 10-weeks intervention, the performances for both Sensory Integration and Gross Motor Skills were improved for both Control group and Experimental group. Table 2 summarizes the test results for the two categories.

**Table 2:** Means and standard deviations of the results of sensory integration and gross motor skills.

	**Control group (*****n*** = **20)**		**Experimental group (*****n*** = **20)**	
	**Pre-intervention**	**Post-intervention**	**Pre-intervention**	**Post-intervention**
**Sensory integration**				
Vestibular	28.90 ± 3.37	29.05 ± 3.22	29.90 ± 3.92	32.30 ± 3.45
Tactile	29.00 ± 2.58	29.15 ± 2.41	29.20 ± 3.49	31.40 ± 2.76
Proprioception	28.65 ± 3.92	28.80 ± 3.75	28.30 ± 4.34	32.05 ± 2.98
**Gross motor skill**				
Balance beam walking speed (s)	16.30 ± 0.39	14.72 ± 0.48	16.32 ± 0.33	11.17 ± 0.25
Single-leg standing time (s)	6.00 ± 0.43	6.54 ± 0.45	6.17 ± 0.29	8.06 ± 0.42
Tennis ball throw (m)	4.66 ± 0.39	5.10 ± 0.33	4.68 ± 0.38	6.94 ± 0.41
Two-legged jump speed (s)	10.03 ± 0.43	9.48 ± 0.50	10.03 ± 0.32	8.05 ± 0.51
10-m shuttle (s)	13.44 ± 0.17	13.31 ± 0.15	13.43 ± 0.21	11.46 ± 0.18

### Sensory integration

#### Vestibular function

Table 2 illustrates the means and standard deviations of Vestibular Function. A 2 × 2 (Group × Test Phase) ANOVA revealed a significant main effect for Test Phase, *F*_(1,38)_ = 52.18, *p* < 0.0001, η^2^ = 0.579, but no significant main effect for Group, *F*_(1,38)_ = 3.78, *p* = 0.059, η^2^ = 0.091. Importantly, the interaction effect was significant, *F*_(1,38)_ = 40.62, *p* < 0.0001, η^2^ = 0.517. *Post hoc* analysis using Tukey's HSD indicated that the Experimental Group outperformed the Control Group significantly in the Post-intervention test phase, with no significant difference between groups observed in the Pre-intervention phase.

#### Tactile

Table 2 presents the means and standard deviations of Tactile function. A 2 × 2 (Group × Test Phase) ANOVA showed a significant main effect for Test Phase, *F*_(1,38)_ = 70.53, *p* < 0.0001, η^2^ = 0.65, but no significant main effect for Group, *F*_(1,38)_ = 1.91, *p* = 0.175, η^2^ = 0.048. The interaction effect was significant, *F*_(1,38)_ = 53.67, *p* < 0.0001, η^2^ = 0.586. *Post hoc* analysis using Tukey's HSD revealed that the Experimental Group significantly outperformed the Control Group in the Post-intervention test, while no significant difference was found between groups in the Pre-intervention test.

#### Proprioception

Table 2 displays the means and standard deviations of Proprioception. A 2 × 2 (Group × Test Phase) ANOVA indicated a significant main effect for Test Phase, *F*_(1,38)_ = 23.09, *p* < 0.0001, η^2^ = 0.378, and no significant main effect for Group, *F*_(1,38)_ = 1.66, *p* = 0.205, η^2^ = 0.042. The interaction effect was significant, *F*_(1,38)_ = 19.67, *p* < 0.0001, η^2^ = 0.341. *Post hoc* analysis using Tukey's HSD showed that the Experimental Group performed significantly better than the Control Group in the Post-intervention test, with no significant difference observed between groups in the Pre-intervention test.

### Gross motor performance

#### Balance beam walking speed

Table 2 presents the means and standard deviations of the Balance Beam task. A 2 x 2 (Group x Test Phase) ANOVA indicated significant main effects for both Group, *F*_(1,38)_ = 355.24, *p* < 0.0001, η^2^ = 0.898, and Test Phase, *F*_(1,38)_ = 2588.74, *p* < 0.0001, η^2^ = 0.986. Importantly, the interaction effect was also significant, *F*_(1,38)_ = 729.08, *p* < 0.0001, η^2^ = 0.95. *Post hoc* analysis using Tukey's HSD revealed that the Experimental Group outperformed the Control Group significantly only in the Post-intervention test, with no significant difference observed between the two groups in the Pre-intervention test.

#### Single-leg standing

Table 2 illustrates the means and standard deviations of Single-Leg Standing. The 2 × 2 (Group × Test Phase) ANOVA showed significant main effects for both Group, *F*_(1,38)_ = 57.03, *p* < 0.001, η^2^ = 0.60, and Test Phase, *F*_(1,38)_ = 411.89, *p* < 0.0001, η^2^ = 0.916. The interaction effect was also significant, *F*_(1,38)_ = 127.10, *p* < 0.0001, η^2^ = 0.77. Further analysis using Tukey's HSD demonstrated that the Experimental Group significantly outperformed the Control Group only in the Post-intervention test, with no significant difference between groups in the Pre-intervention test.

#### Tennis ball throw

Table 2 displays the means and standard deviations of the Tennis Ball Throw task. The 2 × 2 (Group × Test Phase) ANOVA indicated significant main effects for both Group and Test Phase, *F*_(1,38)_ = 68.81, *p* < 0.001, η^2^ = 0.684, and *F*_(1,38)_ = 483.31, *p* < 0.0001, η^2^ = 0.927, respectively. The interaction effect was also significant, *F*_(1,38)_ = 218.56, *p* < 0.0001, η^2^ = 0.852. Subsequent Tukey's HSD analysis highlighted that the Experimental Group excelled significantly over the Control Group post-intervention, while no notable differences emerged in the pre-intervention phase.

#### Two-legged jump speed

Table 2 demonstrates the means and standard deviations of the Two-Legged Jump task. Results of the 2 × 2 (Group x Test Phase) ANOVA reflected significant main effects for both Group and Test Phase, *F*_(1,38)_ = 36.83, *p* < 0.001, η^2^ = 0.479, and *F*_(1,38)_ = 282.81, *p* < 0.0001, η^2^ = 0.882, respectively. The interaction effect was also significant, *F*_(1,38)_ = 81.83, *p* < 0.0001, η^2^ = 0.708. Further investigation using Tukey's HSD affirmed that the Experimental Group surpassed the Control Group significantly in the Post-intervention test, with no significant disparity observed in the Pre-intervention test.

#### 10-m shuttle

Table 2 exhibits the means and standard deviations of the 10-m Shuttle task. Analytical outcomes from the 2 × 2 (Group × Test Phase) ANOVA unveiled significant main effects pertaining to Group and Test Phase, *F*_(1,38)_ = 329.48, *p* < 0.0001, η^2^ = 0.897, and *F*_(1,38)_ = 1,799.01, *p* < 0.0001, η^2^ = 0.979, respectively. Notably, the interaction effect was significant, *F*_(1,38)_ = 1,391.65, *p* < 0.0001, η^2^ = 0.973. *Post hoc* analysis via Tukey's HSD pinpointed superior performance by the Experimental Group post-intervention compared to the Control Group, while preintervention differences were negligible.

## Discussion

Given the high prevalence of CSISP among the Chinese population, there is an urgent need to develop and implement effective intervention strategies to address this condition. The primary objective of this study was to evaluate the effectiveness of integrating basketball training with traditional sensory integration therapy (SIT) in managing CSISP among Chinese children with moderate CSISP.

The current study found that both the traditional SIT group and the group that combined traditional SIT with basketball training showed significant improvements in sensory integration and gross motor skills after the 10-week intervention. However, the group that combined traditional SIT with basketball training demonstrated superior gains compared to the traditional SIT group alone. This combined approach was particularly effective in enhancing vestibular, tactile, and proprioceptive functions, as well as improving gross motor skills such as balance, coordination, and agility.

These results suggest that incorporating physical activities such as basketball into traditional therapeutic approaches can offer a more comprehensive and effective method for addressing the complex challenges associated with CSISP. By integrating these complementary interventions, it is possible to achieve better treatment outcomes and improve the overall quality of life for children with Sensory Integration Disorder. These results are consistent with previous research emphasizing the advantages of integrating physical activities with traditional SIT in addressing CSISP (Xu et al., 2019; Han, 2014; Zhao, 2020; Ge et al., 2019). However, it is important to note that the previous studies primarily focused on integrating individual events and closed motor skills such as taekwondo (Xu et al., 2019), martial arts (Han, 2014), rhythmic rope skipping (Zhao, 2020), and cheerleading (Ge et al., 2019).

The 10-week basketball training program was more effective than the standard sensory integration training program, likely due to its unique components. The dribbling exercises, requiring precise ball control and rhythm, significantly enhanced children's vestibular sensory capabilities. Continuous ball contact further developed tactile abilities, while basketball's group dynamics fostered communication, social skills, and self-confidence. Activities like shooting improved self-esteem, and the competitive nature of the sport built resilience. The blend of static and dynamic exercises also enhanced spatial memory, motor control, and proprioception, underscoring basketball's holistic benefits for sensory integration.

In contrast, the present study makes a notable contribution to the development of the field by demonstrating the potential of incorporating team sports and open motor skills, such as basketball, to enhance both sensory integration and gross motor skills in children with CSISP. Unlike previous research, which primarily explored the effects of integrating individual events and closed motor skills, our study highlights the unique advantages of open motor skills in enhancing both sensory integration and gross motor skills. The team-based nature of basketball fosters social development, promoting communication and collaboration among peers, as supported by Eime et al. (2013), who linked team sports participation to improved self-esteem, reduced social isolation, and enhanced social acceptance.

Research consistently indicates that open motor skills, involving dynamic and unpredictable environments, are superior to closed motor skills for improving children's executive function (Feng et al., 2023; Heilmann et al., 2022). Specifically, open motor skills have been shown to surpass closed motor skills in enhancing processing speed and cognitive abilities (Yongtawee and Woo, 2017), and in improving inhibitory control—the ability to suppress inappropriate but prepotent actions within a given context (Wang et al., 2013). Similarly, studies such as Moreau et al. (2015) and Gökçe et al. (2021) report that open motor skills, such as free-style wrestling and fencing, provide greater cognitive and physical benefits than closed motor skills, such as treadmill running or swimming. Given these findings, it is not surprising that this study observed superior outcomes when integrating basketball training with traditional SIT compared to using traditional SIT alone in improving both sensory integration and gross motor skill performance.

Furthermore, basketball's dynamic and unpredictable nature offers a wealth of sensory stimuli that significantly enhance sensory integration and gross motor skills. The sport involves constant visual tracking of the ball, teammates, and opponents, which improves visual attention, depth perception, and the ability to rapidly shift focus. Proprioceptive feedback is provided through activities like dribbling and shooting, which help children develop body awareness and coordination. Additionally, handling the basketball involves varying textures and pressures, refining tactile sensitivity and hand-eye coordination. The integration of these sensory inputs during the game helps children with CSISP develop more efficient sensory processing pathways. The sport's requirement to adapt to sudden changes in direction, speed, and opponent behavior promotes flexible sensory processing and improves overall motor coordination. The complex movement patterns, such as running, jumping, and dribbling, enhance gross motor skills, balance, and agility. Furthermore, the need for quick decision-making and rapid reactions supports cognitive and motor skill integration, fostering better motor planning and execution. Overall, the multifaceted sensory and motor demands of basketball make it a valuable activity for promoting sensory integration and enhancing gross motor skills in children with CSISP.

Additionally, the social dimension inherent in team sports like basketball offers valuable opportunities for communication and collaboration. These essential interpersonal skills are particularly crucial for children with CSISP, as they provide a structured yet flexible environment for the development of social competencies and the cultivation of selfconfidence. The team-oriented aspect of basketball fosters a sense of community and shared goals, which can be particularly beneficial for children with CSISP. The collaborative and social interactions inherent in team sports like basketball may contribute to the holistic development of children, addressing not only their sensory and motor challenges but also their social and emotional wellbeing. This study underscores the importance of integrating diverse and engaging activities, such as open motor skills, into traditional sensory integration therapy. By expanding the scope of therapeutic approaches, this integrative strategy holds the potential to improve treatment outcomes and enhance the quality of life for children with CSISP.

In addition to demonstrating the effectiveness of integrating basketball training with traditional sensory integration therapy, this study highlights the cultural relevance of this approach within the Chinese context. Basketball enjoys widespread popularity and enthusiasm among children in China, which adds significant value to the research findings. The cultural significance of basketball in Chinese society underscores its relevance and appeal as part of therapeutic interventions, particularly for children diagnosed with CSISP. The deep-rooted passion for basketball in Chinese culture means that children are likely to be both familiar with and enthusiastic about participating in basketball-based activities. This cultural connection can enhance engagement and sustained interest in the therapeutic process, which is crucial for effective intervention. By incorporating a sport that children find enjoyable and culturally resonant, the therapy not only becomes more engaging but also reduces resistance to treatment and fosters a positive, proactive attitude toward therapy. Furthermore, integrating a popular sport like basketball into therapeutic settings helps bridge the gap between clinical environments and everyday activities. This alignment with cultural interests facilitates the application of therapeutic benefits to real-life contexts, enriching the overall therapeutic experience and potentially leading to more effective outcomes and improved quality of life for children with CSISP.

In summary, basketball's emphasis on social interaction, communication, and teamwork supports the development of key social skills in children with CSISP, fostering emotional and cognitive growth. This open, team-based motor skill activity not only aids sensory integration but also offers a holistic approach to improving physical and social functioning, setting it apart from the more limited benefits of closed motor skills. Integrating basketball with traditional SIT aligns with Ayres' Sensory Integration Theory, which highlights the brain's capacity to reorganize in response to sensory inputs, addressing challenges children with CSISP face in processing sensory information.

Basketball, as a dynamic sport, stimulates multiple sensory systems simultaneously. Movements like running, jumping, and dribbling engage the vestibular and proprioceptive systems, enhancing balance, spatial orientation, and body awareness. Additionally, basketball requires executive functions (attention, planning, decision-making), supporting sensory integration through real-time adaptation to stimuli.

Previous research (King et al., 2007; Yogman et al., 2018) has demonstrated the positive impact of integrating play-based interventions into therapy sessions for children facing diverse developmental challenges. These studies underscore the pivotal role of motivation in pediatric rehabilitation and underscore the significance of incorporating activities that are meaningful and enjoyable for children. They suggest that aligning therapy with children's interests can enhance motivation, adherence to treatment plans, and ultimately lead to better outcomes. Therefore, by leveraging children's existing interest in basketball, the combination of basketball training with traditional SIT becomes not only a therapeutic intervention but also a source of enjoyment and fulfillment. Engaging children in activities they find inherently enjoyable can foster a positive attitude toward therapy and promote sustained participation, ultimately leading to more effective outcomes.

### Limitations of the current study

While our study highlights the effectiveness of combining basketball training with traditional SIT, it is important to acknowledge several limitations of our study. Notably, we did not directly compare the efficacy of basketball training—an example of open motor skills—with other closed motor skills, such as gymnastics or track and field, when combined with SIT. As a result, we cannot determine whether basketball training provides superior benefits over other types of physical activities. Future research should address this gap by comparing the effects of various open and closed motor skills activities in conjunction with SIT. Such studies would help clarify the relative advantages of different motor skills training approaches and refine intervention strategies to optimize outcomes for children with CSISP.

Additionally, the duration and intensity of the intervention in our study may have influenced the observed outcomes. A longer follow-up period and a larger sample size would strengthen the evidence regarding the long-term effectiveness of the intervention. Further, examining individual differences—such as age, and severity of CSISP symptoms—could provide insights into how to tailor interventions more effectively to meet the specific needs of each child. By investigating these factors and continuing to integrate diverse therapeutic approaches, we can enhance treatment effectiveness and improve the overall quality of life for children with CSISP.

## Conclusion

In conclusion, our study adds valuable insights to the growing body of literature on the effectiveness of integrating physical activities with traditional Sensory Integration Therapy for treating children with CSISP. Our findings demonstrate that incorporating basketball training—a form of open motor skills training—can serve as a highly beneficial adjunct to traditional SIT. This combination not only enhances sensory integration but also significantly improves gross motor skills in children with CSISP.

The dynamic nature of basketball introduces a diverse array of sensory stimuli and motor challenges that contribute to improved sensory processing and motor coordination. Additionally, the social and team-oriented aspects of basketball foster essential communication and collaborative skills, which are particularly advantageous for children with CSISP. These interactions help build social competencies and self-confidence, further supporting the holistic development of these children.

## Data Availability

The original contributions presented in the study are included in the article/Supplementary material, further inquiries can be directed to the corresponding authors.
