# Supplementary material for: Improving sensory integration in Chinese children with moderate sensory integration challenges through engaging basketball training
Source: Front Psychol. 2025 Jan 15;15:1481945. doi: 10.3389/fpsyg.2024.1481945 (PMC11775159; doi:10.3389/fpsyg.2024.1481945)
Supplement: Supplementary file 1 [file Data_Sheet_1.pdf]

Sensory Integration Course Design

Session	Exercise Name	Exercise Content
1	1. Joyful Ball Pit	Guide the child to jump or step gently into the ball pit. Hide the entire body in the pit and accept the pressure from the balls. Move or swing limbs, body, and neck to adjust gravity perception.
	2. Rolling Big Dragon Ball	Have the child lie on their stomach or back on the ground. Place a large dragon ball on them and roll it slowly back and forth or side to side, or apply gentle pressure. For sensitive children, pressing the back (prone) is easier to accept than pressing the abdomen (supine). Try pressing the feet to help coordinate the brain and body.
2	1. Rolling with Peanut Ball	Have the child lie on their stomach or back. Roll a large peanut ball from their feet to head and back repeatedly. Apply gentle pressure, especially to the neck, ankles, and knees.
	2. Dodgeball	Use massage balls of different sizes to practice throwing and catching with the child, repeating multiple times. After the child adapts, throw a large massage ball to observe their dodging and collision responses. Ensure throws are gentle to avoid injury.
3	1. Slope Rolling	Create a slope with soft blocks at about a 20-degree angle. Let the child lie on a mat and roll down. Guide them to coordinate their hands, feet, and head while rolling.
	2. Cylinder Time Tunnel	Use plastic, metal cylinders, or breathable fabric tunnels that can be entered from both sides. Let the child crawl in head-first or feet-first, guiding them to use their limbs and body effectively.
4	1. Rolling Here and There	Have the child enter a rolling barrel with arms open to protect the head. Gently rotate the barrel, keeping the speed slow. Communicate with the child, stopping immediately if they feel uncomfortable, and help them exit.
	2. Lying on a Balance Board	Have the child lie on a balance board, with limbs naturally extended. Rock side to side with a rhythm to stimulate gravity perception and awaken the brain.
5	1. Wobbling Balance Board	Due to the high center of gravity, remind the child to sit properly and use their hands to maintain balance.
	2. Balance Board Cooperation	Stand on the balance board with the child, holding hands to maintain balance. Practice with the guide leading first, then move together at the same speed, maintaining rhythm.
6	1. Standing on a Balance Board	Let the child stand on the balance board while the guide rocks it slowly. Observe how the child uses their head, torso, hands, and feet to maintain balance.
	2. Rocking and Throwing	Have the child stand on the balance board and rock. Place boxes or baskets 2 meters in front and let the child throw balls into them.

Session	Exercise Name	Exercise Content
7	1. Sitting on a Spinning Top	Have the child sit on a spinning top, with the guide spinning it at about one rotation every two seconds. Do not spin too fast and observe the child's reactions. As they get accustomed, let them spin themselves.
	2. Spinning Top + Throwing	Have the child sit on the spinning top and throw balls into fixed targets while being spun by the guide.
8	1. Sliding Down	Slide down while holding a doll or into a ball pit or simple obstacle to add fun.
	2. Sliding Down Backwards	Slide down head-first in a prone position, which can be scarier. Place a soft mat at the bottom for safety.
9	1. Circle Rolling	Bind three swim rings or tires together, or use a cylindrical roller. Have the child lie horizontally inside and assist with rolling. Avoid injury to the head and hands. Stand 30 cm from the ring and push the ball forward, competing to see who rolls the ball fastest and straightest.
	2. Fun Ladder	Place a ladder between two platforms (10-20 cm high) and have the child climb across, aiding hand and foot coordination. Have them step through the ladder's rungs, improving spatial awareness and body control.
10	1. Activity Rolling Tube (1)	Have the child enter three tied swim rings or tires. For scared children, start with gentle side-to-side rocking, then proceed to rolling.
	2. Activity Rolling Tube (2)	Assist the child in rocking the tube back and forth, then have them stand on the upright tube, maintaining balance with legs apart and arms extended.
11	1. Single-Leg Kicking	Sit on a one-legged chair, balancing with the buttocks and legs. Practice lifting crossed legs. Kick a ball to the wall and let it bounce back.
	2. Hugging the Circle	Use a swim ring or tire suspended from the ceiling, allowing it to rotate 360 degrees. Guide the child to hold the outer ring with hands and feet, maintaining balance. For safety, place a trampoline or soft mat below. Add counting games while swinging to increase fun and confidence.
12	1. Lying on the Circle	Adjust the suspension height so the child's feet do not touch the ground when lying. Observe how they stretch and contract their limbs and muscles.
	2. Circle + Kicking Targets	Have the child sit inside the ring, aiming and kicking targets while swinging. Practice muscle contraction and coordination, maintaining a controlled posture.

Session	Exercise Name	Exercise Content
13	1. Cradle Rocking	Create a cradle by suspending a fabric sling. Let the child lie in it and rock side to side. Add large 360-degree rotations and have the child close their eyes to feel balance.
	2. Web Cable Swinging	Suspend a fabric sling vertically from the ceiling, about 20 cm above the ground. Have the child lie prone or curled up inside, guiding them to lift their head. Assist with rocking back and forth or in circles. Let the child stack blocks or create shapes while swinging.
14	1. Standing in the Web Cable	Suspend the sling about 10 cm above the ground. Have the child sit or stand inside, holding the edges to maintain balance.
	2. Four-Legged Balance on Sling	Place a target 3 meters away. Push the sling quickly forward, and have the child hit the target with a stick. Alternatively, let the child lie prone in the sling, picking up balls or blocks while moving.
15	1. Rocking Horse	Let the child sit or lie on a flat rocking horse (10-20 cm high). Guide them to rock back and forth or side to side 30-100 times.
	2. Jumping on the Trampoline	Have the guide jump with the child on a trampoline to reduce fear. Parent-child activities strengthen their bond. Practice 90-degree and 180-degree turns. Lie the child on the trampoline and bounce by jumping nearby.
16	1. Web Cable Swinging	Suspend the fabric sling vertically from the ceiling, about 20 cm above the ground. Let the child lie prone or curled up inside, guiding them to lift their head. Assist with rocking back and forth or in circles.
	2. Standing in the Web Cable	Suspend the sling about 10 cm above the ground. Have the child sit or stand inside, holding the edges to maintain balance.
17	1. Trampoline Fun	The guide and child each hold one end of a hula hoop and jump on a trampoline. Take turns jumping, guiding the child's visual focus. Play catch or run around the trampoline to enhance tracking skills.
	2. Trampoline + Hand-Eye Coordination	Stand on separate trampolines and play catch. Add skipping ropes or suspend a net for ball throwing games, boosting confidence by comparing scores.
18	1. Kangaroo Jump	Line up and enter a jump sack, holding it up while jumping. Guide the child to feel body control with eyes closed. Jump to a flag and back.
	2. Log Horse Swing	Lie prone on a log horse, holding tightly with hands and feet. Guide gentle rocking. Sit at the ends, gripping with legs, and rock.
19	1. Log Horse Fancy Games	Sit on a log horse, hitting targets with a stick. For safety, place mats below.
	2. Spinning Wheel Swing	Sit on a spinning wheel, gripping with legs and hands. Guide rocking.

Session	Exercise Name	Exercise Content
20	1. Elephant Crawl	Place hand and foot prints on the ground. Form a line and crawl by lifting hips high. Change the print layout for a new challenge.
	2. Scooter Board Games	Lie prone on a scooter board, lifting head and chest like an airplane. Crawl forward using hands. Change direction or turn 360 degrees.
