# Supplementary material for: Improving sensory integration in Chinese children with moderate sensory integration challenges through engaging basketball training
Source: Front Psychol. 2025 Jan 15;15:1481945. doi: 10.3389/fpsyg.2024.1481945 (PMC11775159; doi:10.3389/fpsyg.2024.1481945)
Supplement: Supplementary file 2 [file Data_Sheet_2.pdf]

Basketball Enlightenment Training Course Design

Class	Exercise Name	Exercise Content
1	Ball Handling Exercises + Basketball Games	1. Squat and Push Ball: Children squat, place the basketball in front of them, open their fingers, and use their wrists to move the ball left and right, keeping it in front of them. Once they can skillfully control the ball, ask them to look forward and use their fingers and wrists to move the ball around their body.<br>2. Ball Through Tunnel: Children stand with feet slightly wider than shoulders, bend over, slightly bend knees, place the ball in front, and use fingers and wrists to move the ball through their legs in a figure-eight motion. Alternate hands.<br>3. Around the Waist and Back: Feet together, hold the ball in front, pass it around the waist and back, alternating hands.<br>4. Moving Between Legs: Feet slightly wider than shoulders, hold the ball in front, step forward with one foot, pass the ball between the legs to the other hand, alternate steps.
2	Ball Handling Exercises + Basketball Games	1. Throw and Catch: Stand straight with feet shoulder-width apart, throw the ball up and catch it. Start with low throws and gradually increase height. Add difficulty by clapping before catching.<br>2. Dwarf Rolling Ball: Children squat, pretend to be dwarfs, and push the ball forward.<br>3. Roll and Chase: Bend over, slightly bend knees, push the ball forward with fingers and wrists. Can be done as a race.
3	Stationary Dribbling + Basketball Games	1. Low Stationary Dribble: Dribble with one hand beside the body.<br>2. High Stationary Dribble: Dribble with one hand beside the body.<br>3. Passing Game: Pass the ball overhead to the next person, who passes it between their legs to the next, and so on. Race in groups.
4	Stationary Alternating Dribble + Basketball Games	1. Front Stationary Dribble: Dribble in front of the body with one hand.<br>2. Front Alternating Dribble: Dribble in front with both hands.<br>3. Dribble and Steal Game: In groups, dribble within a set area, trying to knock others' balls while protecting your own. If the ball is knocked out or dribbled out, the player is out.
5	Dribbling While Moving + Basketball Games	1. Straight Line Dribble: Dribble back and forth on a straight line, starting slowly to learn control.<br>2. Dribble Through Obstacles: Dribble through markers, gradually increasing number and difficulty.<br>3. Dodgeball: Stand in a circle, teachers outside roll balls in, children must avoid the balls. If hit, they are out.

Class	Exercise Name	Exercise Content
6	Dribbling While Moving + Basketball Games	1. Jogging Dribble: Dribble while jogging.<br>2. Full Speed Dribble: For more advanced children, dribble at full speed.<br>3. Dribble Relay: In groups, dribble back and forth, passing to the next child.
7	Close Distance Chest Pass + Basketball Games	1. Wall Pass: Pass the ball against a marked spot on the wall.<br>2. Partner Pass: In pairs, pass the ball from 2 meters apart.<br>3. Fastest Pass: In two groups, pass around a circle back to the starting person.
8	Long Distance Chest Pass + Basketball Games	1. Partner Pass: In pairs, pass the ball from 4 meters apart.<br>2. Triangle Pass: In groups of three, pass the ball within a triangle, staying within hoops.<br>3. Two Pass, One Steal: In groups of three, two pass while one tries to steal.
9	Review Dribbling and Passing While Moving	1. Review Dribbling While Moving.<br>2. Learn Passing While Moving: In pairs, pass while moving forward.<br>3. Passing Race: In two teams, pass across the court and back, timed.
10	Passing While Moving	1. Speed Up Passing: From walking to running while passing.<br>2. Four-Corner Pass: In groups of four, pass from one corner to the next, moving to the end of the receiving line after passing.
11	Stationary Chest Shooting	1. Chest Shot: Practice without a ball, then with a ball, not aiming at the hoop.<br>2. Basketball Hopping: Hold the ball between knees, hop to the midline, dribble to the other side, shoot, and pass to the next child.
12	Spot Shooting	1. Chest Shot (Bank Shot): Practice shooting from a fixed spot, focusing on technique and using the backboard. Mark spots on the backboard to aim for.
13	Review Passing and Shooting While Moving	1. Review Passing While Moving.<br>2. Learn Shooting While Moving: Combine dribbling and shooting, stopping to shoot.<br>3. Dribble and Shoot: Line up, dribble towards the hoop, stop, and shoot.
14	Shooting While Moving	1. Chase and Shoot: Children chase a thrown ball, dribble, and shoot.<br>2. Catch and Shoot: Pass to the teacher at the free throw line, catch the return, and shoot.
15	Review Shooting from Different	1. Break down layup steps without shooting, then combine steps and shooting.

Class	Exercise Name	Exercise Content
	Distances, Layups	2. Modified "What's the Time, Mr. Wolf?": Children dribble, freeze when the teacher turns, run home when "6 o'clock" is called.
16	Layups	1. Master Layups.<br>2. Layup Relay: In two groups, dribble from the centerline, make a layup, and return. Pass the ball to the next in line.
17	Review Dribbling, Passing, Defense	1. Learn Side Steps.<br>2. Shoulder-to-Shoulder Jump and Shoot: Line up, jump to the end, retrieve a ball, and shoot. Pass to the next child.
18	Review All Skills	1. Review.<br>2. Pull Shirt and Dribble: In a line, the back child holds the front child's shirt, dribbling forward. First to the end wins.
19	Review All Skills, Learn Basic Rules, One-on-One	1. Review.<br>2. Chase and Shoot: In two teams, first child runs, the next rolls the ball, first child retrieves and shoots.<br>3. Learn Basic Rules: Introduce basic basketball rules like out-of-bounds, traveling, double dribble, fouls. 4. Full-Court One-on-One: In pairs, one attacks, one defends, trying to score or steal.
20	Organize 3/3 Teaching Game	1. Game: Two halves, 7 minutes each, 3-minute halftime.<br>2. In groups of three, play by one-on-one rules, emphasizing teamwork and passing to score. Celebrate after scoring to build confidence and team spirit.
